# A computational assessment of pH-dependent differential interaction of T7 lysozyme with T7 RNA polymerase

**DOI:** 10.1186/s12900-017-0077-9

**Published:** 2017-05-25

**Authors:** Subhomoi Borkotoky, Ayaluru Murali

**Affiliations:** Centre for Bioinformatics, School of Life Sciences, Pondicherry University, Puducherry, 605014 India

**Keywords:** T7 lysozyme, T7 RNA polymerase, Molecular dynamics simulation, Docking, Principal component analysis, T-pad analysis

## Abstract

**Background:**

T7 lysozyme (T7L), also known as N-acetylmuramoyl-L-alanine amidase, is a T7 bacteriophage gene product. It involves two functions: It can cut amide bonds in the bacterial cell wall and interacts with T7 RNA polymerase (T7RNAP) as a part of transcription inhibition. In this study, with the help of molecular dynamics (MD) calculations and computational interaction studies, we investigated the effect of varying pH conditions on conformational flexibilities of T7L and their influence on T7RNAP -T7L interactions.

**Results:**

From the MD studies of the T7L at three different pH strengths *viz.* 5, neutral and 7.9 it was observed that T7L structure at pH 5 exhibited less stable nature with more residue level fluctuations, decrease of secondary structural elements and less compactness as compared to its counterparts: neutral pH and pH 7.9. The T-pad analysis of the MD trajectories identified local fluctuations in few residues that influenced the conformational differences in three pH strengths. From the docking of the minimum energy representative structures of T7L at different pH strengths (obtained from the free energy landscape analysis) with T7RNAP structures at same pH strengths, we saw strong interaction patterns at pH 7.9 and pH 5. The MD analysis of these complexes also confirmed the observations of docking study. From the combined *in silico* studies, it was observed that there are conformational changes in N-terminal and near helix 1 of T7L at different pH strengths, which are involved in the T7RNAP interaction, thereby varying the interaction pattern.

**Conclusion:**

Since T7L has been used for developing novel therapeutics and T7RNAP one of the most biologically useful protein in both *in-vitro* and *in vivo* experiments, this *in silico* study of pH dependent conformational differences in T7L and the differential interaction with T7RNAP at different pH can provide a significant insight into the structural investigations on T7L and T7RNAP in varying pH environments.

**Electronic supplementary material:**

The online version of this article (doi:10.1186/s12900-017-0077-9) contains supplementary material, which is available to authorized users.

## Background

The ~17 kDa lysozyme of bacteriophage T7 (T7L) or simply T7 lysozyme, also known as N-acetylmuramoyl-L-alanine amidase or endolysin, is a product of class II gene of T7 bacteriophage genome. Endolysins have a wide array of usage such as antimicrobial agents [1], food safety [2], against phytopathogenic bacteria [3], enzybiotics [4], disinfectants [5] etc. Endolysins have been categorized into four classes: i) glycosidases (muramidase), ii) endopeptidases, iii) amidohydrolases (amidase), and iv) lytic transglycosylases. Endolysins infect both Gram-positive and Gram-negative bacteria; while the former type contains multiple domains, the later one generally represents single-domain globular proteins (15–20 kDa). T7 lysozyme (T7L) falls into the later type of the endolysins. The T7L is a bi-functional protein that cuts amide bonds in the bacterial cell wall and also inhibits transcription by T7 RNA polymerase. It lyses a range of Gram-negative bacteria by hydrolyzing the amide bond between N-acetylmuramoyl residues and the L-alanine of the peptidoglycan layer. The zinc amidase, T7L, has a zinc atom located in the cleft bound directly to three amino acids and a water molecule; however, the presence of zinc is required for amidase activity but not for inhibition of T7RNAP [6, 7].

As levels of T7L rise, transcriptional-inhibited T7RNAP-T7L complexes form. Though this complex can catalyze the synthesis of short RNA molecules, it fails to clear the abortive initiation phase. It has been found that T7L does not bind to the active site of T7RNAP; alternatively, it binds to a remote site. The RNAP interaction domain (amino acids 2–52) of T7L interacts with T7RNAP at portions of its N-terminal domain (amino acids 307, 309–312), finger sub-domain (amino acids 720, 721, 724, 726, 728 and 736) and palm extended foot module (amino acids 844, 850–853 and 855). The binding of lysozyme may induce two types of conformational changes in these portions either by i) altering the orientation of these portions relative to each other as compared to the apo T7RNAP or ii) by hindering possible conformational changes that may be required during various stages of the transcriptional cycle [8].

**Fig. 1:**
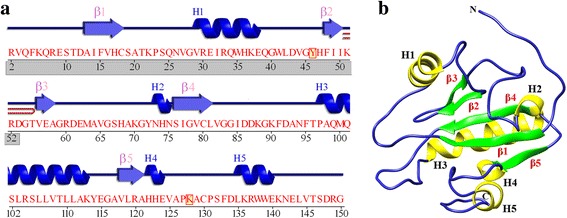
Structural description of T7 lysozyme (PDB ID: 1ARO_L): (**a**) Secondary structure representation of T7L. Helices labeled as H1, H2 etc. whereas beta sheets and beta hairpin are represented by β and . The T7RNAP binding domain is highlighted in the *grey box*. The diagram was generated from PDBsum server; (**b**) three dimensional representation of T7L. Helices are colored in *yellow* and β sheets are colored in *green* and numbered as per their occurrence

T7L has five α-helices and five β-sheets (Fig. 1) and its optimal amidase activity is around pH 7–7.5. This activity decreases to 50% at pH 6.0 and drops significantly with a further decrease in pH with a considerable loss in secondary structural content [7]. Although pH dependent lytic activity of T7L has been studied extensively [7], there is no information on how pH dependence influences the other activity of this bi-functional protein i.e., inhibition of transcription by T7 RNA polymerase. The single subunit polymerase from bacteriophage T7 phage, T7RNAP has a wide array of applications in biological research ranging from over expression of heterologous genes under the control of the T7 promoter [9] to industrial biotechnology [10] and synthetic biology [11]. Due to its advantageous properties, the T7RNAP has been expressed in different environments of prokaryotes to eukaryotes with different organelle (cell nucleus) [12], organs (human liver cell line) [13] etc. Thus a study of pH dependent interaction analysis will help researchers to regulate this biologically important enzyme in low pH environments with the help of mutational studies. It was revealed from a pH based activity profile of wild type T7RNAP [14] that the polymerase has substantial enzymatic activity in the range 7.9 to 9.5, but at a low pH (pH 5) and beyond pH 11 the enzyme exhibited diminished or no activity. Since in this study, our interest was to find out the pH dependent differential interaction of T7 lysozyme with T7 RNA polymerase, we have selected an acidic pH (pH 5) and a basic pH (pH 7.9).

## Methods

### Structure of T7L and T7RNAP

Since the current study is focused on T7RNAP inhibition by T7L, we have used the lysozyme structure from the T7RNAP-T7L crystallographic complex (PDB ID: 1ARO) [8]. The structure of T7RNAP at different pH strengths viz. pH 5, neutral and pH 7.9 were taken from our earlier work [15] (Borkotoky S, Meena CK, Bhalerao GM, Murali A.: An in-silico glimpse into the pH dependent structural changes of T7 RNA polymerase: a protein with simplicity, Manuscript Submitted).

### Molecular dynamics simulation of T7L at different pH strengths

The structure of T7L was subjected to exhaustive molecular dynamics simulation (MDS) up to 40 ns with GROMACS (Groningen Machine for Chemical Simulations) 4.5 simulation package [16] under three different pHs *viz.* 5, neutral and 7.9 using Gromos force field [17]. First, the topologies for each pH strength were generated by setting protonation and deprotonation states of K, R, D, E and H residues as identified by the H++ server [18]. Each system was settled in a cubic box where the edge of the box from the molecule was set to 1.5 nm in all directions. SPC216 water model was used to solvate the box based on periodic boundary conditions. The net charge of the system was maintained for the pH 5.0 and pH 7.9 structure of T7L after protonation and de-protonation step while the neutral pH structure was neutralized by replacing the water molecules with Cl^−^ and Na^+^ counter ions based on their net charge. Each system was minimized by steepest descent algorithm up to a maximum of 50,000 steps and a convergence tolerance of 1000 kJ mol^−1^nm^−1^, following which, conjugate gradient algorithm was used with the same steps and convergence tolerance. For long-range interactions, the PME method was used with a 1.0 nm cut-off. Then, equilibrations were carried out for 100 ps for each system with NVT (constant number of particles, volume, and temperature) with modified Berendsen thermostat with velocity rescaling at 310 K and a 0.1 ps time step, Particle Mesh Ewald coulomb type for long-range electrostatics with Fourier spacing 0.16 followed by NPT (constant number of particles, pressure, and temperature) with Parrinello–Rahman pressure coupling at 1 bar, with a compressibility of 4.5 × 10^−5^ bar^−1^ and a 2 ps time constant. Finally, the equilibrated different pH systems were subjected to 40 ns MD simulation with a time-step of 2 fs. Further analyses of the MD trajectories were carried out using the utilities associated with GROMACS package such as g_rms and g_rmsf to obtain the RMSD (root-mean-square deviation) and the RMSF (root-mean-square fluctuation) values while g_gyrate and do_dssp to calculate the radius of gyration and secondary structure for each time frame.

### Principal component and free energy landscape (FEL) analysis

To describe the high amplitude concerted motion in a trajectory, principal component analysis (PCA) or Essential Dynamics [19, 20] was carried out based on their eigenvectors of the mass-weighted covariance matrix of protein atomic fluctuations. The cosine contents (c_i_) of each principal component (p_i_) of covariance matrix were calculated to generate the free energy landscape defined by PCA analysis. The GROMACS in-built utility “g_covar” was used to generate the covariance matrix using protein backbone as a reference structure for the rotational fit and “g_anaeig” was used to analyze and plot the eigenvectors. Principal components with smaller cosine content values, in general, below 0.2 can yield qualitatively better results with the observation of single basin [21]. Therefore, the first 6 principal components of T7L simulation trajectory at each of different pH strengths were extracted and analyzed based on their cosine values. The cosine content was calculated with “g_analyze” utility. FEL was constructed using cosine contents lesser than 0.2 of the first two projection eigenvectors (defined as PC1 and PC2 respectively). The minimum energy structure extracted from the minimum energy basins of the FELs were used for further analyses.

### Cluster analysis

The RMSD-based structural clustering was performed by the tool g_cluster within GROMACS. The backbone atoms were used in the clustering using GROMOS algorithm [22] to extract clusters of similar conformations. The central structure of the cluster was picked as the representative.

### T-pad analysis

T-pad [23] is a tool which can effectively calculate the intrinsic plasticity of protein residues, along with the occurrence of transitions between distinct residue conformations. This information regarding residue-wise flexibility of the protein and backbone conformational transitions is important for its biological functionality. This analysis calculates protein angular dispersion of the angle ω (PADω) to quantitatively analyze local fluctuations and transitions of individual residues. PADω is a function of ω (= Φ + ψ), and hence dependent on both Ramachandran angles Φ and ψ (CSΦ and CSψ). Unlike torsion angle Cα − C − N − Cα in a peptide bond (CSω), PADω allows quantitative comparisons among residues and among proteins by keeping a narrow range of ω between 0° and 180°. PADω reads as follows,

$$ \mathrm{PAD}\upomega =\frac{180}{\uppi}\ { \cos}^{-1}\left[\frac{1-\mathrm{CS}\upomega}{1+\mathrm{CS}\upomega}\right] $$

T-pad analysis identifies fluctuations (F), long transitions (T) and short transitions (t). A fluctuation (F) is attributed to the fluctuations along a given direction and those along two separate directions are identified as a transition. The difference in long and short transition depends on PAIω (a function of CSω and the Angular Transition Index ATIω). The transitions having PAIω between 30° and 60° are attributed as long transitions and those between 60° and 90° are identified as short transitions. A detailed theory of T-pad tool has been reported by Caliandro et al. [23]. T-pad tool has been successfully used over time to answer various biological questions [24–26].

Here, T-pad analysis was conducted based on molecular dynamics trajectories to understand the structural transitions of the T7L structure at different pH strengths. The MDS trajectories, for T-pad analysis, at pH 5, neutral and pH 7.9 were prepared by extracting the trajectories corresponding to the minimum energy basins of the FEL using the *trjconv* tool of GROMACS.

### Docking of T7L andT7RNAP at different pH

To study the effect of variable pH strengths on the interactions between T7L and T7RNAP, we have used HADDOCK (High Ambiguity Driven protein-protein DOCKing) server [27] for data-driven biomolecular docking. HADDOCK web server has correctly solved structures of more than 60 biomolecular complexes available in PDB and besides has outperformed in CAPRI (Critical Assessment of Predicted interactions) blind docking experiment [28]. In HADDOCK, experimental data (e.g., from mutagenesis, mass spectrometry or a variety of NMR experiments, residual dipolar couplings (RDCs) or hydrogen/deuterium exchange) are entered in the form of active and passive residues. HADDOCK then converts them into a series of Ambiguous Interaction Restraints (AIR). Docking in HADDOCK was performed in three major steps that involve a rigid-body energy minimization, a semi-flexible refinement in torsion angle space and a final refinement in explicit solvent. From each step, a defined set of best complexes is progressed to next stage [29]. The docked complexes are ranked on the basis of the sum of electrostatics, van der Waals, and AIR energy terms. The docking was performed using the structures of T7L obtained from the previous step at different pH strengths and T7RNAP from our earlier work (Borkotoky S, Meena CK, Bhalerao GM, Murali A.: An in-silico glimpse into the pH dependent structural changes of T7 RNA polymerase: a protein with simplicity, Manuscript Submitted). The best-docked complexes were selected based on internal energy complex and binding energy from HADDOCK energies and binding affinities ΔG (kcal/mol) and dissociation constants K_d_ (M) calculated from PRODIGY server [30]. This method uses a simple but robust descriptor of binding affinity based only on structural properties of a protein–protein complex (a combination of the number of contacts at the interface of a protein–protein complex and on properties of the non-interacting surface). The accuracy of this method can be verified by its observed Pearson’s Correlation coefficient of 0.73 between the predicted and measured binding affinities on the benchmark described by Kastritis *et al.*[31]. To confirm these results, the docked complexes were further subjected to MD simulation.

### Molecular dynamics simulation of T7L and T7RNAP complexes

The complexes of T7L and T7RNAP obtained from the docking step were subjected to molecular dynamics simulation (MDS) up to 60 ns with GROMACS 4.5 simulation package to observe the stability of the docked complexes. Since both the constituents of the complexes were obtained from exhaustive MDS studies and are the minimum energy conformations at respective pH values, protonation was not considered and total charge was neutralized by adding ions to each system. Rest of the MDS protocol was kept same as that of T7L. The number of hydrogen bonds between the complexes was calculated using the g_hbond utility of GROMACS.

## Results

### Molecular dynamics simulations of T7L

In order to study the pH effect on T7L, we performed MD simulations in different pH conditions using GROMACS. To measure the conformational stability of the proteins after 40 ns of simulations at pH values 5, neutral and 7.9 various utilities were used.

**Fig. 2:**
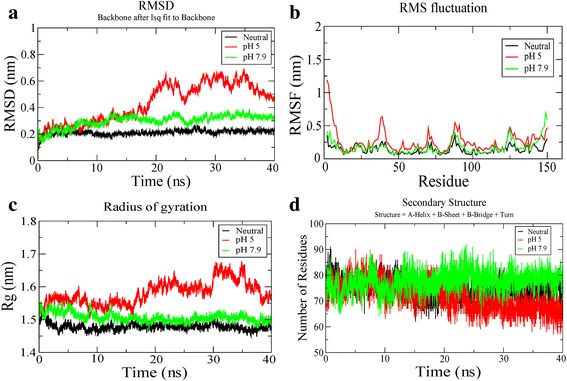
MD simulation results of T7 Lysozyme at three different pH strengths viz. neutral, pH 7.9 and pH 5: (**a**) RMSD plots of simulated trajectories, (**b**) RMSF plots of the simulated trajectories, (**c**) Radius of gyration plot of the simulated trajectories, (**d**) DSSP plot of the simulated trajectories

The RMSD (Root Mean Square Deviation) profile (Fig. 2a) for backbone residues showed that the RMSD, during the 40 ns of simulation period, exhibited stabilization at 0.3 and 0.2 nm for pH strengths of 7.9 and neutral respectively, whereas, at pH 5, it exhibited comparatively increased deviation with RMSD close to 0.7 nm.

The RMSF (Root Mean Square Fluctuation) profiles were also calculated for T7L at different pH strengths and are shown in Fig. 2b. From the RMSF plot, it can be seen that the overall fluctuations of the protein were maximum for pH 5 among the three pH values simulated. At pH 5, it was noticed that the N-terminal (residues 2–7) showed the highest fluctuations in the range 0.6 to 1.2 and other significant fluctuations were observed near helix 1 (residues 38–40). Compared to the fluctuations seen at pH 5, these residues showed considerably lower fluctuations in the range of 0.2 to 0.4 nm at other two pH strengths. However, in the C-terminal region (residues 49–50), T7L showed stronger fluctuations (in the range of 0.6–0.7 nm) at pH 7.9 compared to other two pH strengths.

The plot for Rg (radius of gyration) variation during the simulation time (Fig. 2c) showed that the maximum values for pH strength of 5 were close to 1.7 nm whereas for pH 7.9 and neutral it was close to 1.5 nm. The radius of gyration is a parameter used as an indicator to determine the compactness of the protein and the lowest Rg value corresponds to compactness. Hence, we can say that the compactness of T7L structure decreases at pH 5.

The secondary structure profile (Fig. 2d) of the simulated trajectories showed that there is a decrease of secondary structure at pH 5. This observation is in agreement with the recent circular dichroism (CD) spectroscopy profiles [7], wherein it was reported a decrease in structural content with a decrease in pH strength.

### Principal component and free energy landscape analysis

**Fig. 3:**
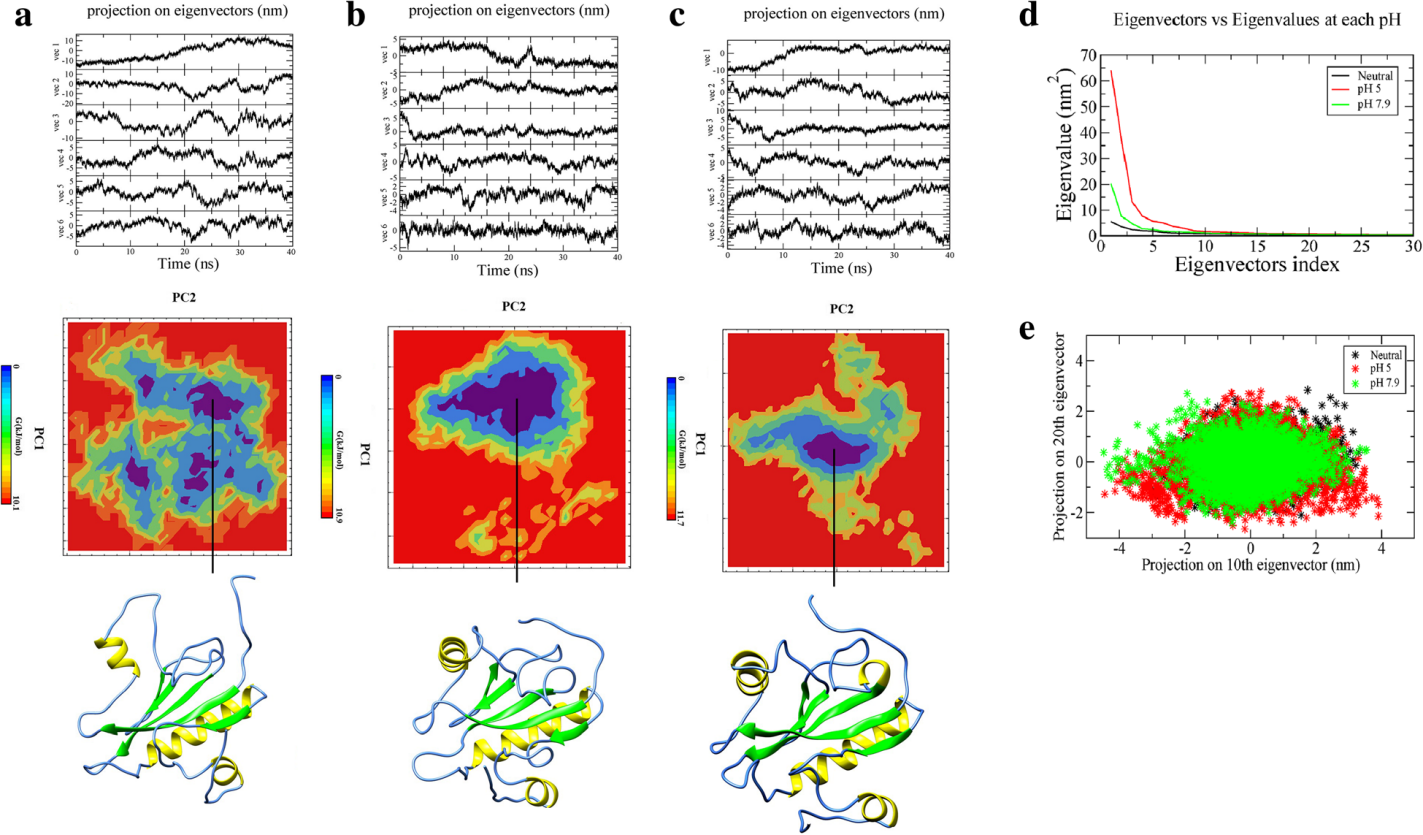
PCA analyses of 40 ns simulation trajectories of T7L at three different pH values depicting the motions along the first six eigenvectors and FEL analyses of T7L depicting low energy basin along with the representative structure retrieved at each pH: (**a**) pH 5, (**b**) Neutral pH and (**c**) pH 7.9. Secondary structure color scheme is same as Fig. 1. **d** Eigenvalues for T7L at each pH, shown in decreasing order of magnitude and obtained from the backbone coordinate covariance matrix as a function of the eigenvector index. **e** The projections of the trajectory onto the planes defined by the 10^th^ and 20^th^ eigenvectors from the protein coordinate covariance matrix for each pH

Generally, the first ten eigenvectors represent most of the motions that illustrate the relevant combined motions within an atomic system [24]. We retrieved the first five, the tenth, and the twentieth projections from the protein trajectories at each pH during 40 ns simulation and projected them onto the eigenvectors as obtained from the covariance matrices (Fig. 3). Steep curves of eigenvalues were obtained after plotting eigenvalues against the eigenvectors at each pH (Fig. 3d), and it was observed that 90% of the backbone motion is covered by the first 20 eigenvectors. These results indicate that the motions of the backbone reached their equilibrium fluctuations in the first ten eigenvectors. The trajectories were projected onto the planes defined by two eigenvectors (the tenth and twentieth eigenvectors) from the backbone coordinate covariance matrix for each pH (Fig. 3e). A strong correlation was observed between the projections of these two eigenvectors onto the plane of the backbone motion at each pH, and they filled the expected ranges almost completely which indicates that there is no high projection observed far from the diagonal. This clearly supports that the MD simulation time interval used to extract the trajectories are sufficient for the FEL graph generation and further analysis. The principal components (PCs), extracted with cosine content closer to 0.2, PC1 and PC2, for individual trajectories at different pH strengths, were used to construct the FEL contour maps (Fig. 3). The contour map at pH 5 (Fig. 3a) showed multiple energy clusters depicting the structural transition to distinct active conformational states. These multiple clusters are due to the fluctuation of N-terminal region which is otherwise more stable at other two conditions (also can be seen in RMSF plot). The coordinates from FEL map with minimum energy cluster (at 26 ns) were used to retrieve the low energy representative structure. On the other hand, the FEL maps at neutral (Fig. 3b) and pH 7.9 (Fig. 3c) showed only one energy minimum at 39 ns and 28 ns, and lowest energy representative structures corresponding to the coordinates at 39 ns and 28 ns were retrieved respectively.

### Cluster analysis

**Fig. 4:**
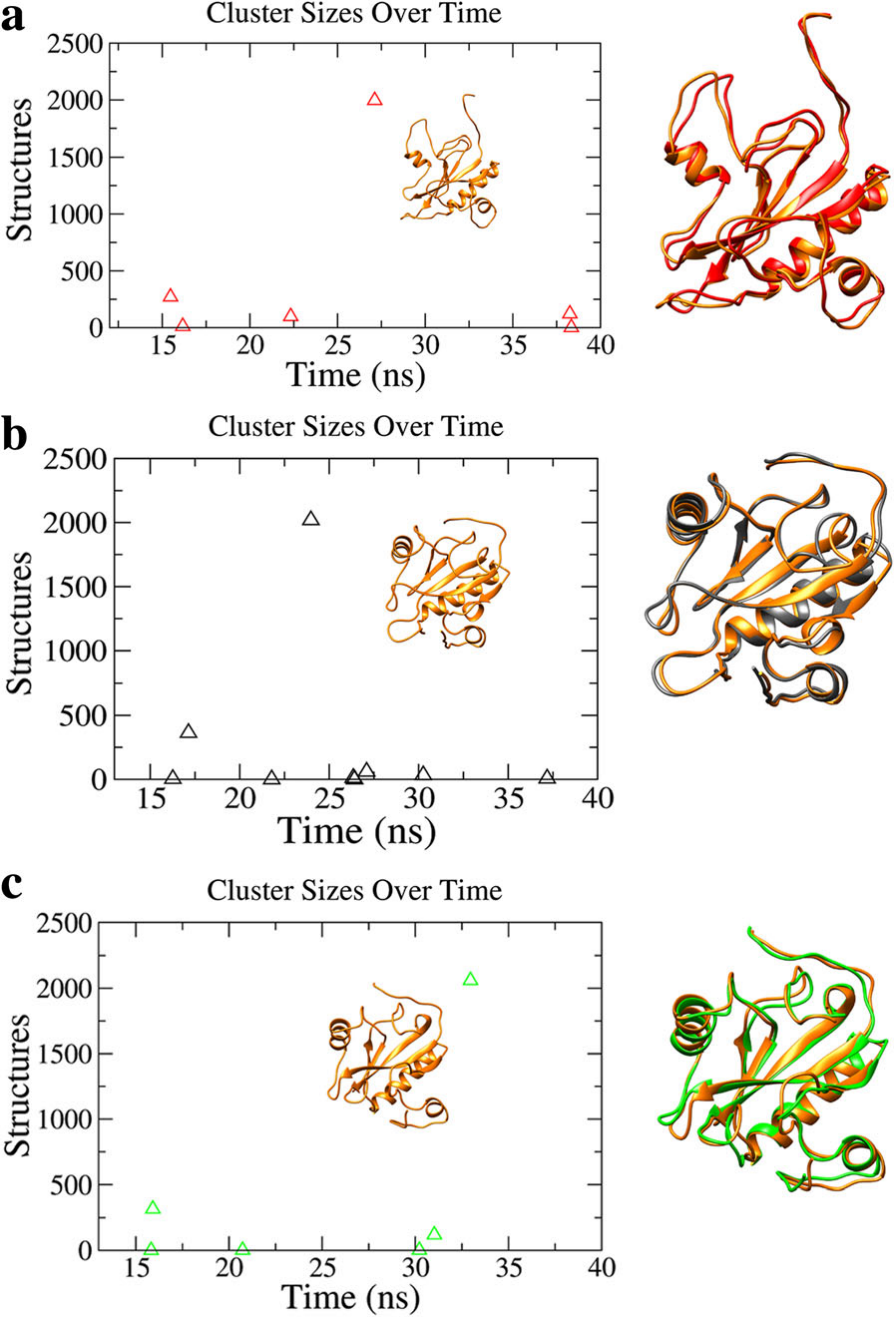
The number of cluster at each pH, (**a**) pH 5, (**b**) pH 7 and (**c**) pH7.9. The representative central structures from each of the top cluster at each pH were shown in *orange* color in each graph. On the *right* panel the central structures were aligned with the FEL representatives at each pH (*colored red*, *grey* and *green* for pH 5, neutral and pH 7.9 respectively)

Cluster analysis combined with FEL analysis allows us to establish whether a correlation exists between structural similarity and minimum energy basins in the sampled trajectories. As differences appear at RMSD graph at about 15 ns, with clear differences between 20 ns and 30 ns, the first 15 ns of the trajectory were not used to determine the average structures. Different RMSD cutoffs were adopted for cluster analysis at each pH i.e., the average RMSD values derived from each RMSD matrices (Additional file 1) were selected (for pH 5, cutoff: 0.33 nm, pH 7, cutoff: 0.13 nm, pH 7.9, cutoff: 0.16 nm). The central structure of the highly populated cluster was picked at each pH (Fig. 4). The central structures obtained from the trajectories at pH 5 (at 27 ns), neutral (at 24 ns) and pH 7.9 (at 32 ns) were represented by clusters having the highest number of structures and each representative structure was superimposed with the representative structures obtained from FEL analysis (Fig. 4). It was observed that the structures were highly similar and the time frames used to extract the structures from FEL were also represented by the top clusters obtained from the cluster analysis (Additional file 1). Hence we used the FEL derived structures for further analysis.

### Structural transition in T7L

To get into the detail of structural transitions of T7L at different pH strengths, we attempted to understand the local fluctuations and conformational transitions using respective MD trajectories.

**Fig. 5:**
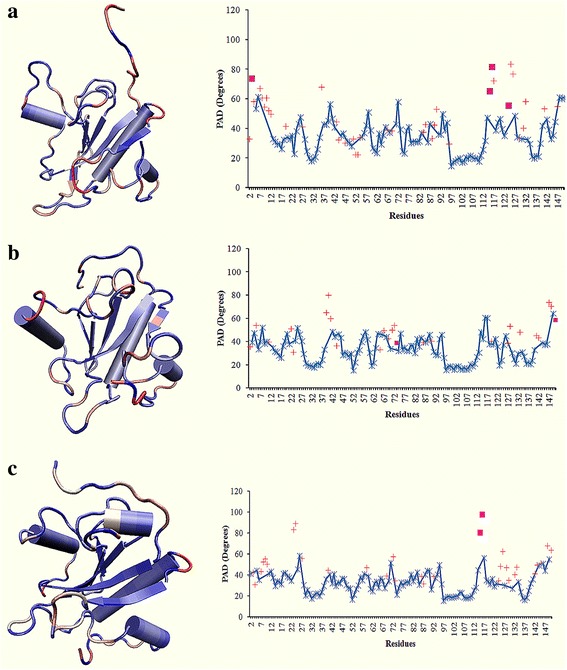
The T-PAD results with their PAD degrees from MD simulation at (**a**) pH 5, (**b**) neutral and (**c**) pH 7.9. The three dimensional representations in the *left* panel are colored *blue* to *red* in order of their increasing PAD degrees. On the *right* panel, the local fluctuations represented as fluctuations (), transitions () and short transitions () were plotted against the residues

At pH 5, the N-terminal residues (2–7) that showed the highest fluctuations in the RMSF plot were checked for their local fluctuations. The residues Arg 2 (33.2°), Gln 4 (58.4°), Gln 7 (67.1°) showed transitions, Val 3 (73.9°) showed short transition and residues Phe 5 and Lys 6 showed fluctuations with PAD degrees 53° and 61° (Fig. 5a). At neutral pH, Arg 2 (36.2°) and Phe 5 (54.3°) showed transitions, Val 3 and Gln 4 showed fluctuations with PAD degrees 39.7° and 47.8° and residue Lys 6 and Gln 7 showed fluctuations with PAD degrees 33.9° and 40.5° (Fig. 4b). At pH 7.9, Arg 2, Phe 5 and Lys 6 showed fluctuations with PAD degrees 41.4°, 44.3° and 35.7° while Val 3 and Gln 4 and Gln 7 showed transitions with PAD degrees 42.9°, 31.15° and 43.5° respectively (Fig. 4c).

Other residues identified with significant transitions at pH 5 are Ala 117 (72.3°), Val 125 (83.7°) and Ala 126 (77°). Whereas, short transitions are found at residues Glu 115 (65.3°) and Gly 116 (81.7°); and highest fluctuations were found in Asp 148 (61.2°) (Fig. 5a).

At neutral pH (Fig. 5b), highest transitions are found at residues Gln 39 (65.5°), Gly 40 (80.4°), Trp 41 (60.3°), Ser 147 (74.2°) and Asp 148 (83.8°). The highest short transition was found at Gly 150 (58.9°) and top fluctuations are found in Gly 116 (60.8°), Ala 117 (60.7°) and Arg149 (64.4°).

At pH 7.9 (Fig. 4c), highest transitions are found at residues Pro 23 (83.8°), Ser 24 (89.5°), Ala (62.9°), Asp 148 (68.3°), and Gly 150 (72.3°). The residues showing highest PAD degrees of short transitions are Glu 115 (97.86°) and Gly116 (80.7°); and the highest fluctuation was found at Asn 26 with PAD degree 58.3°.

### T7L and T7RNAP interactions at different pH

After docking the representative structures of T7RNAP at different pH strengths with T7 lysozyme in HADDOCK server, the best interaction models were selected based on HADDOCK score and energies (internal energy complex and binding energy). The complex of T7RNAP with T7L was noticed to have fair internal and binding energy in comparison to those of other counterparts (Table 1). These complexes were further submitted for PRODIGY analysis to calculate binding affinity (ΔG) and dissociation constant (K_d_). The complex at pH 7.9 showed strongest binding affinity with ΔG = −12.5 kcal/mol and better dissociation constant 7.1e–10 M. The complexes at pH 5 and neutral pH were observed to have lesser affinity −12.2 kcal/mol and −10.0 kcal/mol respectively, whereas dissociation constants were found to be 1.2e–09 M and 5.0e–08 M. The lysozyme complex with pH 7.9 representative forms 12 H-bonds and 179 non-bonded contacts and 4 salt bridges. While for pH 5 and neutral pH, 12 and 14 H-bonds were observed respectively (Additional files 2, 3 and 4). The number of non-bonded contacts was found to be 149 and 150 for complexes at pH5 and neutral pH respectively, while the number of salt bridges was found to be 1 and 5 respectively. At all three pH values, lysozyme was seen to be interacting with the N-terminal domain, finger sub-domain and extended foot module. These results indicate that the conformational changes occurred at T7 RNAP and T7L at pH 7.9 are suitable for strong interaction; on the other hand, at lower pH, the attained conformations do not contribute to a strong interaction. Details of the interactions are included in the additional files 1, 2 and 3.

**Table 1:** HAADOCK docking results for the complexes of T7RNAP and T7L at different pH conditions. The HADDOCK energies are in arbitrary units (a.u.). Binding affinities and dissociation constants are calculated from PRODIGY server

Complex (T7RNAP + Lys)	HADDOCK Energies		Number of interactions		PRODIGY Analysis	
	Internal energy complex	Binding energy	H-bond	Non bonded	Binding affinity ΔG (kcal/mol)	Dissociation constant Kd (M)
pH 5	−41913	−58488	12	149	−12.2	1.2e-09
Neutral	−42364	−58007	14	150	−10.0	5.0e-08
pH 7.9	−42435	−55193	12	179	−12.5	7.1e-10

### Stability analysis of T7L and T7RNAP complexes

**Fig. 6:**
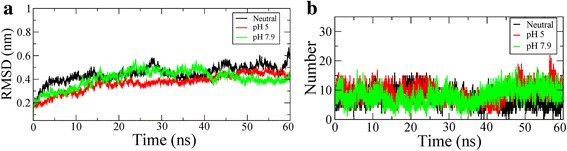
MD simulation results of T7L and T7RNAP complexes at three different pH strengths viz. neutral, pH 7.9 and pH 5: (**a**) RMSD plot of simulated trajectories, (**b**) Number of H-bond plot of the T7RNAP-T7L complex

From the 60 ns MDS study of the T7L-T7RNAP complexes obtained from the earlier step, we saw that the complex at pH 7.9 demonstrated a stable dynamics after approximately 40 ns time period than the other two counterparts (Fig. 6a). The complex at pH 5 was found to be more stable than the neutral counterpart after 40 ns. These RMSD patterns confirm the docking results (Table 1) where we saw similar patterns in binding affinities and dissociation constants. The number of hydrogen bonds (H-bond) formed between the complexes (Fig. 6b) showed that after 40 ns both the complexes at pH 7.9 and pH 5 have increased the number than the neutral pH counterpart, thereby contributing to the higher stability as seen in the RMSD plot.

## Discussion

**Fig. 7:**
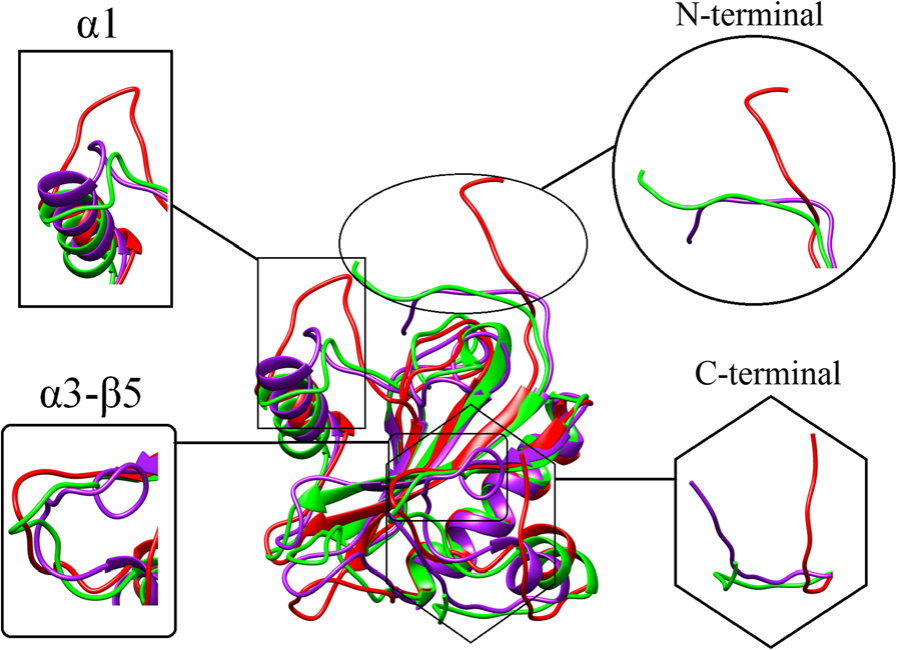
Comparison of the T7L representatives at three pH strengths: pH 5 (*red*), neutral (*purple*) and pH 7.9 (*green*). Different regions with strong conformational change are highlighted in the insets

We conducted the current study to gain clearer insights into the residue level differences in the T7L structure at different pH strengths and how the changes affect the interaction with T7RNAP. The MD simulation studies at different pH strengths showed that the lysozyme structure at pH 7.9 and neutral pH are stable in comparison to pH 5, with higher residue level fluctuations at low pH prominently at N-terminal region and near α-helix 1 (H1). The number of secondary structure elements of the T7L was observed to be decreased in lower pH (pH 5) in agreement with the experimental results [7]. We have also observed a compactness of the structure of T7L under pH 7.9 and neutral pH compared to pH 5. The multiple low energy basins of the FEL of pH 5 trajectory (Fig. 3) in comparison of the single low energy basins at neutral and pH 7.9 also showed the overall stability of T7L at higher pH. From PCA and FEL analysis, we obtained the minimum energy representative structures at different pH strengths. These structures (Fig. 7) are also in agreement to the radius of gyration plot (Fig. 2c), where an open conformation was observed at pH 5. This observation was linked to the loss of secondary structure content at pH 5 (Fig. 2d). The RMSD based clustering analysis also shown strong agreement to FEL analysis.

As we have seen a flipping out movement in the N-terminal region (Fig. 3a), we investigated the N-terminal end (residues Arg 2-Gln 7) showing high fluctuations in the RMSF plot (Fig. 2b) for local fluctuations and conformational transitions. From the T-pad analysis (Fig. 5) it can be proposed that the short transition at Val 3, transition at the two glutamines at positions 4 and 7 and fluctuation at Lys 6 are responsible for the flipping out nature as evidenced by the high PAD degrees in the range of 61°–74° (Fig. 7). The possible mechanism for closed conformation of the N-terminal loop at pH 7 is that it was maintained via a series of hydrophobic interactions between the N-terminal loop and the loop connecting β3 and H2 (L_3,2_). Here, the L_3,2_ contains both polar and non-polar amino acids which are not stimulated by their protonation state; hence the aliphatic side chains of L_1,2_ form a series of hydrophobic interactions (Additional file 5). In case of the free energy representative structure of T7L at pH 5.0, the protonation and de-protonation states of Arg 2, Lys 6, Arg 8, Glu 9 and Asp 12 (2-RVQFKQRESTDA-13) and the residues Arg 60, Asp 61, Glu 62, His 68 and Lys 70 of L_3,2_ region (59-GRDEMAVGSHAKGY-72) repel the N-terminal orientation and displaced away from the L_3,2_ region with the loss of hydrophobic interactions with N-terminal residues Val 2 and Phe 5. While in the case of the free energy representative structure of T7L at pH 7.9, Phe 5 maintained the interaction which prevented it from complete displacement.

Another conformational variation observed near helix-1 at pH 5 was due to the loss of helical content from residues His 36 to Gln 39. These residues were also observed with transitions and fluctuations with higher PAD degrees in a range of 42°–68° as compared to the PAD degree ranges at neutral pH (20°–65°) and pH 7.9 (20°–36°). Though the region between helix-3 and β sheet- 5 (residues Glu 115- Ala 117) showed high PAD degrees they did not contribute any conformational difference neither loss of secondary structural content at all the three pH. This observation can be explained by the maintained hydrophobic interactions by Ala 117 with Ile 14 and Tyr 114 in all three pH representatives (Additional file 5). The residues Val 125 and Ala 126 near helix-4 showed strong transitions with PAD degrees 83.7° and 77° at pH 5 as compared to other counterparts at neutral and pH 7.9 with PAD degree range 37°–45° and 48°–63° respectively with minor conformational differences. The C-terminal residues Asp 148 to Gly 150 also showed high PAD degrees at pH 5 in the range of 60° to 61°, at neutral 71° to 59° and at pH 7.9 the range was 55° to 68° with conformational differences. In this case we observed that, the hydrophobic interaction made by the C-terminal residue Leu 144 with residues of the loop connecting β4 and H3 (L_4,3_) and within H3 at pH 7 (Leu 144 : Ala 93, Leu 144 : Phe 95 and Leu 144 : Met 100) and pH 7.9 (Leu 144 : Pro 97 and Leu 144 : Met 100); while these interactions were absent at pH 5 driving the C-terminal away from L_4,3_ (Additional file 5)_._

**Fig. 8:**
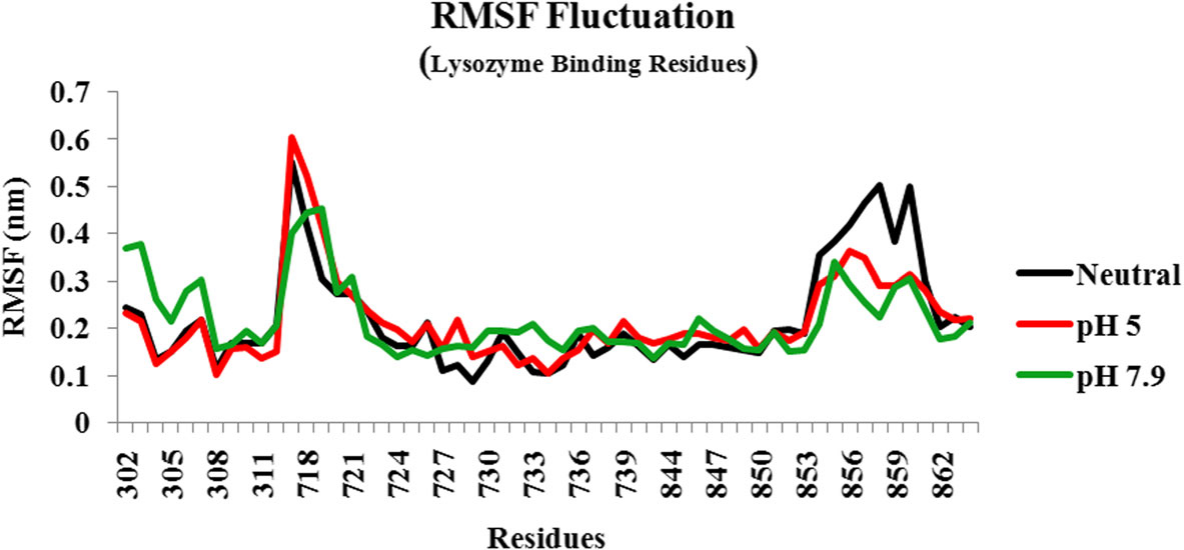
Fluctuations of lysozyme binding residues of T7RNAP before binding of T7L

Upon docking the individual representatives of T7L and T7RNAP at different pH strengths, it was observed that the T7L interacts with T7RNAP more strongly at both pH 5 and pH 7.9 rather than neutral pH with pH 7.9 representative having a higher K_d_ value. This observation was further clarified by MD simulation of all the three complexes and it was noted that the complexes at pH 5 and pH 7.9 are more stable than neutral counterpart, with pH 7.9 complex being most stable. The complexes at pH 5 and pH 7.9 also maintained the number of H-bonds, while a decrease in number was observed in the case of the neutral complex. From the MD study of T7L, we found that the T7L structure forms an open/relaxed structure at pH 5. The pH 7.9 representative also shows a comparatively relaxed conformation than neutral pH. Due to these differences, T7L is making differential interactions with T7RNAP at their individual pH. Hence, it can be proposed that the structural changes observed at both pH 5 and pH 7.9 in T7L as well as T7RNAP (Fig. 8) (Borkotoky S, Meena CK, Bhalerao GM, Murali A.: An in -silico glimpse into the pH dependent structural changes of T7 RNA polymerase: a protein with simplicity, Manuscript Submitted) are favorable for the interaction of both proteins.

## Conclusion

Change in physiological environments such as pH, temperature etc. are integral to structure function relationship of proteins. The present study successfully identified pH dependent structural changes in T7 lysozyme, complementing experimental studies but with a more residue level information. The MD simulation and docking studies showed that, though T7L has poor amidase activity at low pH, it does not hamper the T7RNAP interaction ability. The results obtained here can be used for mutational studies for modification of the T7L structure to control levels of inhibition of T7RNAP as well as other structural studies of related bacteriophage amidases such as T3, K11 etc.

## Additional files

Additional file 1: RMSD matrices of the trajectories at a) pH 5, b) pH 7 and c) pH 7.9; Distribution of cluster ids were represented along the trajectories at d) pH 5, e) pH 7 and f) pH 7.9 at a function of time. (DOCX 1258 kb)

Additional file 2: HADDOCK docking results of T7RNAP and Lysozyme (at pH 5). A surface representation of the docked complex is shown. (DOCX 276 kb)

Additional file 3: HADDOCK docking results of T7RNAP and Lysozyme (at neutral pH). A surface representation of the docked complex is shown. (DOCX 274 kb)

Additional file 4: HADDOCK docking results of T7RNAP and Lysozyme (at pH 7.9). A surface representation of the docked complex is shown. (DOCX 331 kb)

Additional file 5: Hydrophobic interactions within 5 angstroms for T7L representative structures at individual pH: a) pH 7, b) pH 7.9 and c) pH 5. The interactions were calculated by Protein Interactions Calculator (PIC) server (http://pic.mbu.iisc.ernet.in). (DOCX 979 kb)

## References

[1] Nelson DC, Schmelcher M, Rodriguez-Rubio L, Klumpp J, Pritchard DG, Dong S, Donovan DM (2012). Endolysins as antimicrobials. Adv Virus Res.

[2] Schmelcher M, Loessner MJ (2016). Bacteriophage endolysins: applications for food safety. Curr Opin Biotechnol.

[3] de Vries J, Harms K, Broer I, Kriete G, Mahn A, During K, Wackernagel W (1999). The bacteriolytic activity in transgenic potatoes expressing a chimeric T4 lysozyme gene and the effect of T4 lysozyme on soil- and phytopathogenic bacteria. Syst Appl Microbiol.

[4] Tiwari R, Dhama K, Chakrabort S, Kapoor S. Enzybiotics: new weapon in the army of antimicrobials: a review. Asian J Anim Vet Adv. 2014;9(3):144–163.

[5] Hoopes JT, Stark CJ, Kim HA, Sussman DJ, Donovan DM, Nelson DC (2009). Use of a bacteriophage lysin, PlyC, as an enzyme disinfectant against Streptococcus equi. Appl Environ Microbiol.

[6] Cheng X, Zhang X, Pflugrath JW, Studier FW (1994). The structure of bacteriophage T7 lysozyme, a zinc amidase and an inhibitor of T7 RNA polymerase. Proc Natl Acad Sci U S A.

[7] Sharma M, Kumar D, Poluri KM (2016). Elucidating the pH-Dependent Structural Transition of T7 Bacteriophage Endolysin. Biochemistry-Us.

[8] Jeruzalmi D, Steitz TA (1998). Structure of T7 RNA polymerase complexed to the transcriptional inhibitor T7 lysozyme. EMBO J.

[9] Studier FW, Moffatt BA (1986). Use of bacteriophage T7 RNA polymerase to direct selective high-level expression of cloned genes. J Mol Biol.

[10] Kortmann M, Kuhl V, Klaffl S, Bott M (2015). A chromosomally encoded T7 RNA polymerase-dependent gene expression system for Corynebacterium glutamicum: construction and comparative evaluation at the single-cell level. Microb Biotechnol.

[11] Shis DL, Bennett MR (2014). Synthetic biology: the many facets of T7 RNA polymerase. Mol Syst Biol.

[12] Dunn JJ, Krippl B, Bernstein KE, Westphal H, Studier FW (1988). Targeting bacteriophage T7 RNA polymerase to the mammalian cell nucleus. Gene.

[13] Aoki Y, Aizaki H, Shimoike T, Tani H, Ishii K, Saito I, Matsuura Y, Miyamura T (1998). A human liver cell line exhibits efficient translation of HCV RNAs produced by a recombinant adenovirus expressing T7 RNA polymerase. Virology.

[14] Osumi-Davis PA, Sreerama N, Volkin DB, Middaugh CR, Woody RW, Woody AY (1994). Bacteriophage T7 RNA polymerase and its active-site mutants. Kinetic, spectroscopic and calorimetric characterization. J Mol Biol.

[15] Borkotoky S, Meena CK, Murali A (2016). Interaction analysis of T7 RNA polymerase with heparin and its low molecular weight derivatives - an in silico approach. Bioinf Biol Insights.

[16] Pronk S, Pall S, Schulz R, Larsson P, Bjelkmar P, Apostolov R, Shirts MR, Smith JC, Kasson PM, van der Spoel D (2013). GROMACS 4.5: a high-throughput and highly parallel open source molecular simulation toolkit. Bioinformatics.

[17] Oostenbrink C, Villa A, Mark AE, van Gunsteren WF (2004). A biomolecular force field based on the free enthalpy of hydration and solvation: the GROMOS force-field parameter sets 53A5 and 53A6. J Comput Chem.

[18] Gordon JC, Myers JB, Folta T, Shoja V, Heath LS, Onufriev A (2005). H++: a server for estimating pKas and adding missing hydrogens to macromolecules. Nucleic Acids Res.

[19] Amadei A, Linssen AB, Berendsen HJ (1993). Essential dynamics of proteins. Proteins.

[20] Burkoff NS, Varnai C, Wells SA, Wild DL (2012). Exploring the energy landscapes of protein folding simulations with Bayesian computation. Biophys J.

[21] Maisuradze GG, Leitner DM (2007). Free energy landscape of a biomolecule in dihedral principal component space: sampling convergence and correspondence between structures and minima. Proteins.

[22] Daura X, Gademann K, Jaun B, Seebach D, van Gunsteren WF, Mark AE (1999). Peptide folding: when simulation meets experiment. Angew Chem Int Edit.

[23] Caliandro R, Rossetti G, Carloni P (2012). Local fluctuations and conformational transitions in proteins. J Chem Theory Comput.

[24] Topno NS, Kannan M, Krishna R (2016). Interacting mechanism of ID3 HLH domain towards E2A/E12 transcription factor–An Insight through molecular dynamics and docking approach. Biochem Biophysics Rep.

[25] Vadlamudi Y, Muthu K, Kumar MS (2016). Structural exploration of acid sphingomyelinase at different physiological pH through molecular dynamics and docking studies. RSC Adv.

[26] Bafunno V, Bury L, Tiscia GL, Fierro T, Favuzzi G, Caliandro R, Sessa F, Grandone E, Margaglione M, Gresele P (2014). A novel congenital dysprothrombinemia leading to defective prothrombin maturation. Thromb Res.

[27] van Zundert GC, Rodrigues JP, Trellet M, Schmitz C, Kastritis PL, Karaca E, Melquiond AS, van Dijk M, de Vries SJ, Bonvin AM (2016). The HADDOCK2.2 Web server: user-friendly integrative modeling of biomolecular complexes. J Mol Biol.

[28] Gurung AB, Das AK, Bhattacharjee A (2017). y Disruption of redox catalytic functions of peroxiredoxin-thioredoxin complex in Mycobacterium tuberculosis H37Rv using small interface binding molecules. Comput Biol Chem.

[29] De Vries SJ, van Dijk M, Bonvin AMJJ (2010). The HADDOCK web server for data-driven biomolecular docking. Nat Protoc.

[30] Xue LC, Rodrigues JP, Kastritis PL, Bonvin AM, Vangone A (2016). PRODIGY: a web server for predicting the binding affinity of protein-protein complexes. Bioinformatics.

[31] Kastritis PL, Moal IH, Hwang H, Weng Z, Bates PA, Bonvin AM, Janin J (2011). A structure-based benchmark for protein-protein binding affinity. Protein Sci.

