# Supplementary material for: A computational assessment of pH-dependent differential interaction of T7 lysozyme with T7 RNA polymerase
Source: BMC Struct Biol. 2017 May 25;17:7. doi: 10.1186/s12900-017-0077-9 (PMC5445346; doi:10.1186/s12900-017-0077-9)
Supplement: Supplementary file 1 — RMSD matrices of the trajectories at a) pH 5, b) pH 7 and c) pH 7.9; Distribution of cluster ids were represented along the trajectories at d) pH 5, e) pH 7 and f) pH 7.9 at a function of time. (DOCX 1258 kb) [file 12900_2017_77_MOESM1_ESM.docx]

**Additional File 1**


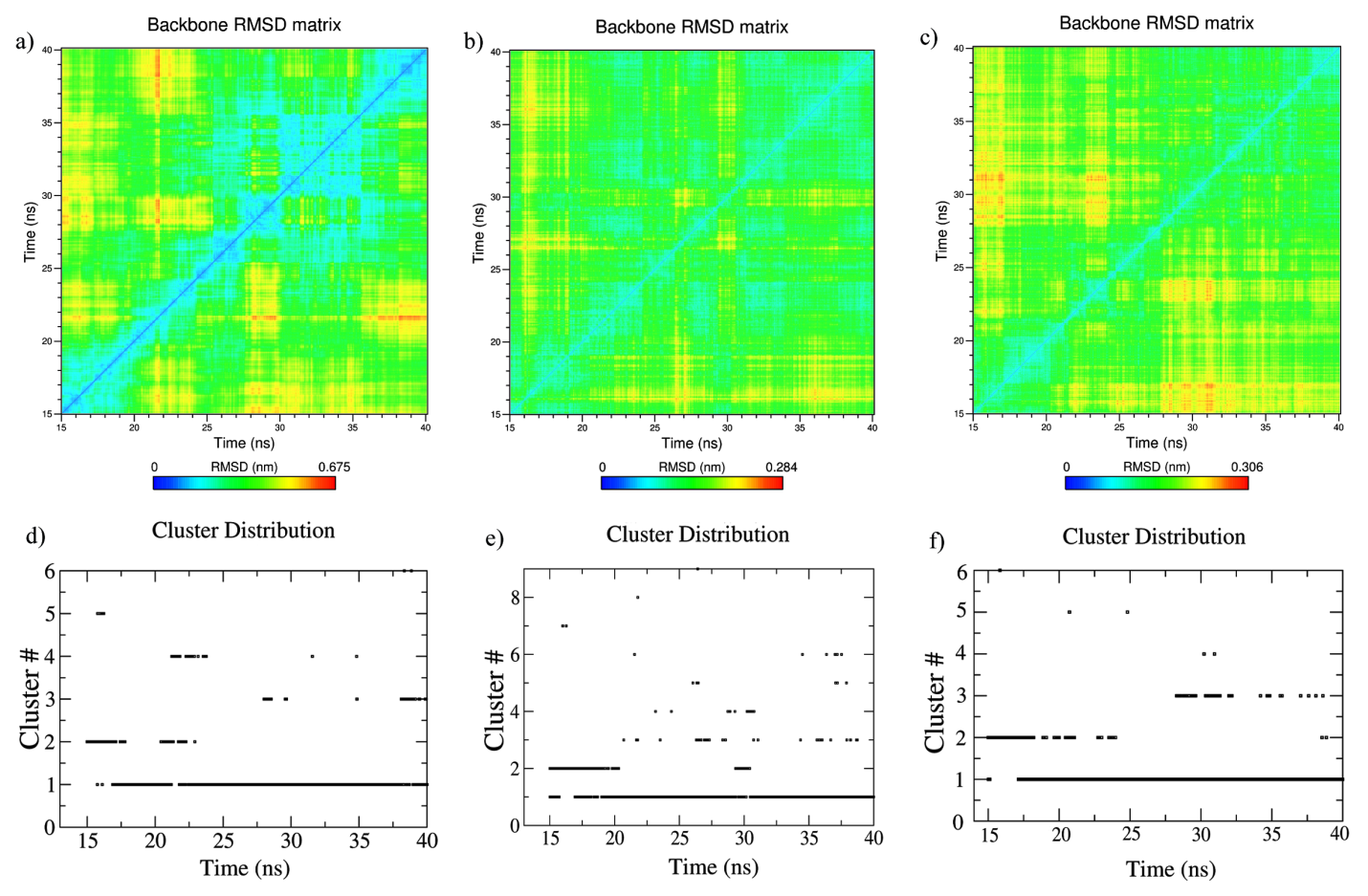


RMSD matrices of the trajectories at a) pH 5, b) pH 7 and c) pH 7.9; Distribution of cluster ids were represented along the trajectories at d) pH 5, e) pH 7 and f) pH 7.9 at a function of time.
