# Supplementary material for: A computational assessment of pH-dependent differential interaction of T7 lysozyme with T7 RNA polymerase
Source: BMC Struct Biol. 2017 May 25;17:7. doi: 10.1186/s12900-017-0077-9 (PMC5445346; doi:10.1186/s12900-017-0077-9)
Supplement: Supplementary file 2 — HADDOCK docking results of T7RNAP and Lysozyme (at pH 5). A surface representation of the docked complex is shown. (DOCX 276 kb) [file 12900_2017_77_MOESM2_ESM.docx]

Additional file 2

HADDOCK docking results of T7RNAP and Lysozyme (at pH 5). A surface representation of the docked complex is shown.

| Complex | Surface representation of the binding mode  ( T7RNAP= blue[Chain A] , Lysozyme= green [Chain B], Binding interface= yellow[T7RNAP] , red [Lysozyme]) |
| --- | --- |
| T7RNAP + Lys | 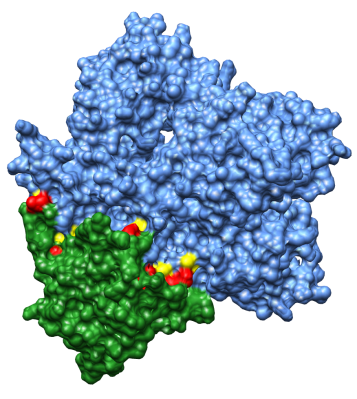 |
| Hydrogen bonds | |
| \| Atom Name. \| Res name \| Res No. \| Chain name \|  \| Atom Name. \| Res name \| Res No. \| Chain name \| Distance(Å) \| \| --- \| --- \| --- \| --- \| --- \| --- \| --- \| --- \| --- \| --- \| \| N \| MET \| 1 \| A \| <--> \| O \| ALA \| 20 \| B \| 2.63 \| \| OG1 \| THR \| 3 \| A \| <--> \| NZ \| LYS \| 22 \| B \| 2.84 \| \| NZ \| LYS \| 303 \| A \| <--> \| ND1 \| HIS \| 36 \| B \| 3.04 \| \| NH1 \| ARG \| 307 \| A \| <--> \| O \| VAL \| 44 \| B \| 2.8 \| \| NH2 \| ARG \| 307 \| A \| <--> \| O \| VAL \| 27 \| B \| 3.27 \| \| NH2 \| ARG \| 307 \| A \| <--> \| O \| GLY \| 28 \| B \| 2.92 \| \| OD2 \| ASP \| 310 \| A \| <--> \| OG \| SER \| 19 \| B \| 3.11 \| \| O \| GLY \| 732 \| A \| <--> \| NZ \| LYS \| 70 \| B \| 2.91 \| \| OD2 \| ASP \| 847 \| A \| <--> \| NH1 \| ARG \| 8 \| B \| 2.65 \| \| OE1 \| GLN \| 848 \| A \| <--> \| NE2 \| HIS \| 74 \| B \| 2.72 \| \| OD1 \| ASP \| 851 \| A \| <--> \| N \| GLN \| 7 \| B \| 2.67 \| \| N \| LEU \| 858 \| A \| <--> \| O \| VAL \| 3 \| B \| 2.93 \| | |
| Salt bridges | |
| \| Atom Name. \| Res name \| Res No. \| Chain name \|  \| Atom Name. \| Res name \| Res No. \| Chain name \| Distance(Å) \| \| --- \| --- \| --- \| --- \| --- \| --- \| --- \| --- \| --- \| --- \| \| OD1 \| ASP \| 847 \| A \| <--> \| NH1 \| ARG \| 8 \| B \| 2.65 \| | |
