# Supplementary material for: A computational assessment of pH-dependent differential interaction of T7 lysozyme with T7 RNA polymerase
Source: BMC Struct Biol. 2017 May 25;17:7. doi: 10.1186/s12900-017-0077-9 (PMC5445346; doi:10.1186/s12900-017-0077-9)
Supplement: Supplementary file 3 — HADDOCK docking results of T7RNAP and Lysozyme (at neutral pH). A surface representation of the docked complex is shown. (DOCX 274 kb) [file 12900_2017_77_MOESM3_ESM.docx]

Additional file 3

HADDOCK docking results of T7RNAP and Lysozyme (at neutral pH). A surface representation of the docked complex is shown.

| Complex | Surface representation of the binding mode  ( T7RNAP= blue[Chain A] , Lysozyme= green [Chain B], Binding interface= yellow[T7RNAP] , red [Lysozyme]) |
| --- | --- |
| T7RNAP + Lys | 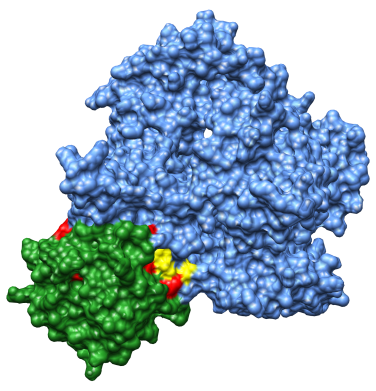 |
| Hydrogen bonds | |
| \| Atom Name. \| Res name \| Res No. \| Chain name \|  \| Atom Name. \| Res name \| Res No. \| Chain name \| Distance(Å) \| \| --- \| --- \| --- \| --- \| --- \| --- \| --- \| --- \| --- \| --- \| \| O \| MET \| 1 \| A \| <--> \| NZ \| LYS \| 22 \| B \| 3.09 \| \| NZ \| LYS \| 303 \| A \| <--> \| OE2 \| GLU \| 38 \| B \| 2.7 \| \| NH1 \| ARG \| 307 \| A \| <--> \| O \| TRP \| 35 \| B \| 2.77 \| \| NH1 \| ARG \| 307 \| A \| <--> \| OE1 \| GLN \| 39 \| B \| 2.84 \| \| NH2 \| ARG \| 307 \| A \| <--> \| OE1 \| GLN \| 39 \| B \| 2.96 \| \| O \| GLU \| 309 \| A \| <--> \| NZ \| LYS \| 22 \| B \| 3.03 \| \| OE2 \| GLU \| 309 \| A \| <--> \| NZ \| LYS \| 22 \| B \| 2.63 \| \| OD1 \| ASP \| 310 \| A \| <--> \| NZ \| LYS \| 22 \| B \| 2.64 \| \| OH \| TYR \| 312 \| A \| <--> \| O \| GLY \| 89 \| B \| 3.04 \| \| OD2 \| ASP \| 731 \| A \| <--> \| NZ \| LYS \| 128 \| B \| 2.66 \| \| NE2 \| GLN \| 848 \| A \| <--> \| OG \| SER \| 67 \| B \| 2.91 \| \| O \| ALA \| 850 \| A \| <--> \| NZ \| LYS \| 6 \| B \| 2.73 \| | |
| Salt bridges | |
| \| Atom Name. \| Res name \| Res No. \| Chain name \|  \| Atom Name. \| Res name \| Res No. \| Chain name \| Distance(Å) \| \| --- \| --- \| --- \| --- \| --- \| --- \| --- \| --- \| --- \| --- \| \| NZ \| LYS \| 303 \| A \| <--> \| OE1 \| GLU \| 38 \| B \| 2.7 \| \| OE2 \| GLU \| 309 \| A \| <--> \| NZ \| LYS \| 22 \| B \| 2.63 \| \| OD2 \| ASP \| 310 \| A \| <--> \| NZ \| LYS \| 22 \| B \| 2.64 \| \| OD2 \| ASP \| 731 \| A \| <--> \| NZ \| LYS \| 128 \| B \| 2.66 \| \| OD1 \| ASP \| 851 \| A \| <--> \| NH1 \| ARG \| 60 \| B \| 2.76 \| | |
