# Supplementary material for: A computational assessment of pH-dependent differential interaction of T7 lysozyme with T7 RNA polymerase
Source: BMC Struct Biol. 2017 May 25;17:7. doi: 10.1186/s12900-017-0077-9 (PMC5445346; doi:10.1186/s12900-017-0077-9)
Supplement: Supplementary file 4 — HADDOCK docking results of T7RNAP and Lysozyme (at pH 7.9). A surface representation of the docked complex is shown. (DOCX 331 kb) [file 12900_2017_77_MOESM4_ESM.docx]

Additional file 4

HADDOCK docking results of T7RNAP and Lysozyme (at pH 7.9). A surface representation of the docked complex is shown.

| Complex | Surface representation of the binding mode  ( T7RNAP= blue[Chain A] , Lysozyme= green [Chain B], Binding interface= yellow[T7RNAP] , red [Lysozyme]) |
| --- | --- |
| T7RNAP + Lys | 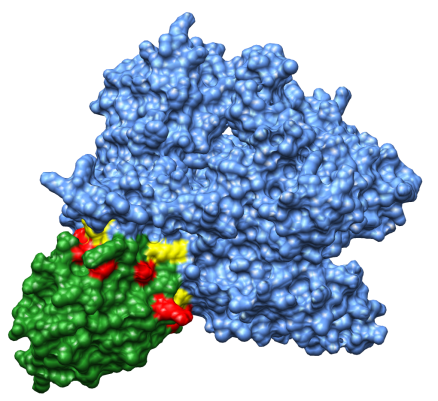 |
| Hydrogen bonds | |
| \| Atom Name. \| Res name \| Res No. \| Chain name \|  \| Atom Name. \| Res name \| Res No. \| Chain name \| Distance(Å) \| \| --- \| --- \| --- \| --- \| --- \| --- \| --- \| --- \| --- \| --- \| \| NZ \| LYS \| 303 \| A \| <--> \| O \| LYS \| 37 \| B \| 2.88 \| \| NH2 \| ARG \| 307 \| A \| <--> \| O \| ARG \| 30 \| B \| 3.1 \| \| O \| ASP \| 310 \| A \| <--> \| NZ \| LYS \| 22 \| B \| 2.8 \| \| OD1 \| ASP \| 310 \| A \| <--> \| NE2 \| GLN \| 25 \| B \| 2.72 \| \| OD1 \| ASP \| 834 \| A \| <--> \| NZ \| LYS \| 88 \| B \| 2.64 \| \| OD1 \| ASP \| 844 \| A \| <--> \| NZ \| LYS \| 90 \| B \| 2.74 \| \| OD1 \| ASP \| 847 \| A \| <--> \| NZ \| LYS \| 70 \| B \| 2.69 \| \| OD1 \| ASP \| 851 \| A \| <--> \| ND2 \| ASN \| 73 \| B \| 2.91 \| \| OD2 \| ASP \| 851 \| A \| <--> \| NE1 \| TRP \| 41 \| B \| 2.93 \| \| ND1 \| HIS \| 854 \| A \| <--> \| ND2 \| ASN \| 73 \| B \| 3.02 \| \| O \| GLU \| 855 \| A \| <--> \| NZ \| LYS \| 6 \| B \| 2.97 \| \| OG \| SER \| 856 \| A \| <--> \| O \| PHE \| 5 \| B \| 2.74 \| | |
| Salt bridges | |
| \| Atom Name. \| Res name \| Res No. \| Chain name \|  \| Atom Name. \| Res name \| Res No. \| Chain name \| Distance(Å) \| \| --- \| --- \| --- \| --- \| --- \| --- \| --- \| --- \| --- \| --- \| \| NE \| ARG \| 307 \| A \| <--> \| OE2 \| GLU \| 38 \| B \| 3.8 \| \| OD1 \| ASP \| 834 \| A \| <--> \| NZ \| LYS \| 88 \| B \| 2.64 \| \| OD1 \| ASP \| 844 \| A \| <--> \| NZ \| LYS \| 90 \| B \| 2.74 \| \| OD2 \| ASP \| 847 \| A \| <--> \| NZ \| LYS \| 70 \| B \| 2.69 \| | |
