# Supplementary material for: A computational assessment of pH-dependent differential interaction of T7 lysozyme with T7 RNA polymerase
Source: BMC Struct Biol. 2017 May 25;17:7. doi: 10.1186/s12900-017-0077-9 (PMC5445346; doi:10.1186/s12900-017-0077-9)

Additional file 5

Hydrophobic interactions within 5 angstroms for T7L representative structures at individual pH: a) pH 7, b) pH 7.9 and c) pH 5. The interactions were calculated by Protein Interactions Calculator (PIC) server (http://pic.mbu.iisc.ernet.in)


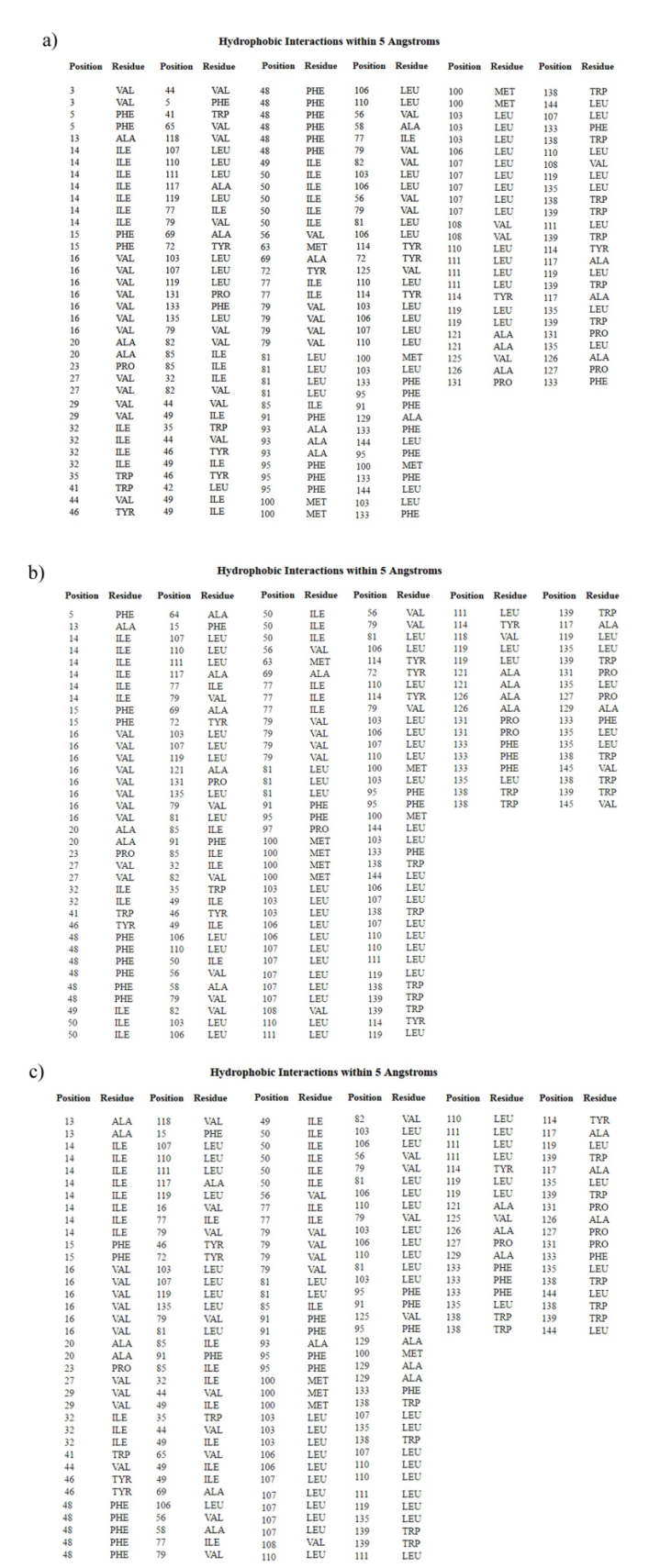

Supplement: Supplementary file 5 — Hydrophobic interactions within 5 angstroms for T7L representative structures at individual pH: a) pH 7, b) pH 7.9 and c) pH 5. The interactions were calculated by Protein Interactions Calculator (PIC) server (http://pic.mbu.iisc.ernet.in). (DOCX 979 kb) [file 12900_2017_77_MOESM5_ESM.docx]
